# Charter of the London University

**Published:** 1837-01

**Authors:** 


### CHARTER OF THE LONDON UNIVERSITY.

IT gives us the greatest pleasure to be able to lay before our readers the Charter of the new Metropolitan University, which has at length received the royal sanction: the Institution may therefore be considered as actually established. The provisions in it relative to the power of conducting examinations, and granting degrees in every department of medical science, possess the utmost interest to all medical men, and promise to be of the greatest advantage to the profession and to the public.

(COPY.)

*Whitehall; Dec.* 1, 1836.

My Lord:—I have the honour to transmit to your lordship the Charter of the University of London.

His Majesty has been pleased to approve of the appointment of your lordship as Chancellor, and of Mr. Lubbock as the first Vice-Chancellor of the University.

I feel confident that it is not necessary to recommend to your lordship either a zealous attention to the interests of learning, or a constant regard to those principles of religious freedom which have furnished motives for the royal grant.

I have no less reliance on the distinguished men who are associated with yourself and Mr. Lubbock in the government of the University.

You may be assured that, on my part also, I shall esteem it an honour to cooperate in the advancement of an Institution destined to confer the distinctions justly due to proficiency in literature, science, or art, without imposing a test of religious opinions, or binding by the fetters of the seventeenth century the talent and merit of the present enlightened age.

I have the honour to be, my Lord, your obedient humble servant,

The Earl of Burlington, &c. J. RUSSELL.

---

William the Fourth, by the grace of God of the United Kingdom of Great Britain and Ireland, King, Defender of the Faith, to all whom these presents shall come, greeting: Whereas, we have deemed it to be the duty of our Royal office, for the advancement of religion and morality and the promotion of useful knowledge, to hold forth to all classes and denominations of our faithful subjects, without any distinction whatsoever, an encouragement for pursuing a regular and liberal course of education; and considering that many persons do prosecute or complete their studies, both in the metropolis and in other parts of our United Kingdom, to whom it is expedient that there should be offered such facilities, and on whom it is just that there should be conferred such distinctions and rewards as may incline them to persevere in these their laudable pursuits. Now know ye, that, for the purpose of ascertaining, by means of examination, the persons who have acquired proficiency in literature, science, and art, by the pursuit of such course of education, and of rewarding them by academical degrees, as evidence of their respective attainments, and marks of honour proportioned thereunto, we do, by virtue of our prerogative Royal, and of our especial grace, certain knowledge, and mere motion, by these presents, for us, our heirs, and successors, will, grant, declare, and constitute

Our right trusty and well-beloved cousin, William Cavendish, Earl of Burlington,

The Right Rev. Father in God Edward Lord Bishop of Durham,

The Right Rev. Father in God William Lord Bishop of Chichester,

Our right trusty and well-beloved Councillor Henry Baron Brougham and Vaux, and

Our trusty and well-beloved George Biddle Airy, Esq., our Astronomer Royal, and Fellow of the Royal Society,

Andrew Amos, Esq., Barrister-at-Law,

Thomas Arnold, Doctor in Divinity,

John Austin, Esq., Barrister-at-Law,

Neil Arnott, Esq., Doctor in Medicine,

John Bacot, Esq., Member of the Royal College of Surgeons,

Francis Beaufort, Esq., Captain in our Royal Navy, Hydrographer of the Admiralty, and Fellow of the Royal Society,

Archibald Billing, Esq., Doctor in Medicine, and Fellow of the Royal College of Physicians,

William Thomas Brande, Esq., Vice-President of the Royal Society,

James Clark, Esq., Doctor in Medicine, Fellow of the Royal Society,

Philip Cecil Crampton, Esq., Doctor of Civil Law, Fellow of the Royal Society, and our Surgeon-General in Ireland,

John Dalton, Esq., Doctor of Civil Law, and Fellow of the Royal Society,

William Empson, Esq., Barrister-at-Law, Professor of General Polity and the Laws of England at the East-India College,

Michael Faraday, Esq., Doctor of Civil Law, Fellow of the Royal Society,

Sir Stephen Love Hammick, Bart., Member of the Royal College of Surgeons,

John Stevens Henslow, Clerk, Master of Arts, Professor of Botany in the University of Cambridge,

Cornwallis Hewett, Esq., Doctor in Medicine, and Downing Professor of Medicine in the University of Cambridge,

Thomas Hodgkin, Esq., Doctor in Medicine,

Francis Kiernan, Esq., Member of the Royal College of Surgeons,

John George Shaw Lefevre, Esq., Fellow of the Royal Society,

Charles Locock, Esq., Doctor in Medicine, one of the Physicians Extraordinary to Her Majesty,

John William Lubbock, Esq., Vice-President of the Royal Society,

Sir James M'Grigor, Baronet, Doctor in Medicine, Doctor of Civil Law, Fellow of the Royal Society, Fellow of the College of Physicians, one of our Physicians Extraordinary, and Director-General of the Army Medical Board,

Richard Rainy Pennington, Esq., Member of the Royal College of Surgeons,

Jones Quain, Esq., Doctor in Medicine,

John Ridout, Esq., Member of the Royal College of Surgeons,

Peter Mark Roget, Esq., Doctor in Medicine, Secretary of the Royal Society,

Nassau William Senior, Esq., one of the Masters of our High Court of Chancery, and Fellow of the Royal Society,

Joseph Henry Jerrard, Doctor of Laws, Principal of the Bristol College,

Richard Sheepshanks, Clerk, Fellow of the Royal Society,

John Sims, Esq., Doctor in Medicine,

Cornop Thirlwall, Clerk, Fellow of Trinity College, Cambridge,

James Walker, Esq., Fellow of the Royal Society, and

Henry Warburton, Esq., Member of the Commons' House of Parliament, and Fellow of the Royal Society,

—during our royal will and pleasure, and all the persons whom we may hereafter appoint to be Chancellor, Vice-Chancellor, or Fellows, as hereinafter mentioned, one body politic and corporate, by the name of the University of London, by which name such body politic shall have perpetual succession, and shall have a common seal, and shall by the same name sue and be sued, implead and be impleaded, and answer and be answered unto in every Court of us, our heirs, and successors. And we do hereby will and ordain, that by the same name they and their successors shall be able and capable in law to take, purchase, and hold to them and their successors any goods, chattels, or personal property whatsoever, and shall also be able and capable in law, notwithstanding the statutes of mortmain, to take, purchase, and hold to them and their successors, not only all such lands, buildings, hereditaments, and possessions, as may be from time to time exclusively used and occupied for the immediate purposes of the said University, but also any other lands, buildings, hereditaments, and possessions whatsoever, situate within our United Kingdom of Great Britain and Ireland, not exceeding the annual value of 10,000*l.*; such annual value to be calculated and ascertained at the period of taking, purchasing, or acquiring the same; and that they and their successors shall be able and capable in law to grant, demise, alien, or otherwise dispose of all or any of the property, real or personal, belonging to the said university, and also to do all other matters incidental or appertaining to a body corporate. And we do hereby further will and ordain, that the said body politic and corporate shall consist of Chancellor, one Vice-Chancellor, and such number of fellows or members of the Senate as we shall from time to time appoint under our sign manual; and that our right trusty and right well-beloved cousin the aforesaid William Cavendish, Earl of Burlington, be the first Chancellor, John William Lubbock, Esq., the first Vice-Chancellor, and the aforesaid Edward Lord Bishop of Durham, William Lord Bishop of Chichester, Henry Baron Brougham and Vaux, George Biddle Airy, Andrew Amos, Thomas Arnold, John Austin, Neil Arnott, John Bacot, Francis Beaufort, Archibald Billing, William Thomas Brande, James Clark, Philip Cecil Crampton, John Dalton, William Empson, Michael Faraday, Sir Stephen Love Hammick, John Stevens Henslow, Cornwallis Hewett, Thomas Hodgkin, Francis Kiernan, John George Shaw Lefevre, Charles Locock, John William Lubbock, Sir James M'Grigor, Richard Rainy Pennington, Jones Quain, John Ridout, Peter Mark Roget, Nassau William Senior, Joseph Henry Jerrard, Richard Sheepshanks, John Sims, Cornop Thirlwall, James Walker, and Henry Warburton, be the first fellows and members of the senate thereof. That whenever a vacancy shall occur in the office of Chancellor of the said university, either by death, resignation, or otherwise, we will, under our sign manual, nominate a fit and proper person to be the Chancellor instead of the Chancellor occasioning such vacancy. That the office of Vice-Chancellor of the said university shall be an annual office; and the Vice-Chancellor hereinbefore named shall, at the expiration of one year from the 1st of July, 1837, go out of office, and the said fellows or members of the Senate shall, at a meeting to be holden by them for that purpose, on some day within a month before the expiration of the tenure of the said office, of which due notice shall be given, elect one other fit and proper person to be the Vice-Chancellor of the said university, and so from time to time annually; or, in case of the death, resignation, or other avoidance of any such Vice-Chancellor, before the expiration of his year of office, shall, at a meeting to be holden by them for that purpose as soon as conveniently may be, of which due notice shall be given, elect some other fit and proper person to be Vice-Chancellor for the remainder of the year in which such death, resignation, or other avoidance shall happen; such person to be chosen from among themselves by the major part of the fellows present at such meeting, and to be approved of by the Chancellor of the said university for the time being.

That we reserve to ourselves to be the visitor of the said University of London, with authority to do all the things which pertain to visitors, as often as to us shall seem meet.

That the Chancellor, Vice-Chancellor, and fellows for the time being, shall have the entire management of, and superintendence over the affairs, concerns, and property of the said university; and in all cases unprovided for by this our charter, it shall be lawful for the Chancellor, Vice-Chancellor, and Fellows, to act in such manner as shall appear to them best calculated to promote the purposes intended by the said University; and the said Chancellor, Vice-Chancellor, and Fellows shall have full power from time to time to make, and also to alter any by-laws and regulations (so as the same be not repugnant to the laws of our realm, or to the general objects and provisions of this our charter) touching the examinations for degrees, and the granting of the same, and touching the mode and time of convening the meetings of the Chancellor, Vice-Chancellor, and Fellows, and in general touching all other matters whatsoever regarding the said university; and all such by-laws and regulations, when reduced into writing, and after the common seal of the said university shall have been affixed thereto, shall be binding upon all persons members thereof, and all candidates for degrees to be conferred by the same, all such by-laws and regulations having been first submitted to one of our principal Secretaries of State, and approved of and countersigned by him.

That all questions which shall come before the Chancellor, Vice-Chancellor, and Fellows, shall be decided by the majority of the members present; and the chairman at any such meeting shall have a vote, and in case of an equality of votes, a second or casting vote.

That no question shall be decided at any meeting unless the Chancellor or Vice-Chancellor, and five Fellows, or, in the absence of the Chancellor and Vice-Chancellor, unless six Fellows at the least shall be present at the time of such decision.

That, at every meeting of the said Chancellor, Vice-Chancellor, and Fellows, the Chancellor, or in his absence the Vice-Chancellor, shall preside as chairman, or in the absence of both, a chairman shall be chosen by the members present, or the major part of them.

That the said Chancellor, Vice-Chancellor, and Fellows for the time being, shall have full power from time to time to appoint, and, as they shall see occasion, to remove all examiners, officers, and servants of the said University.

That once, at least, in every year, the said Chancellor, Vice-Chancellor, and Fellows, shall cause to be held an examination of candidates for degrees; and on every such examination the candidates shall be examined either by examiners appointed for the purpose from among the Fellows by the said Chancellor, Vice-Chancellor, and Fellows, or by other examiners so to be appointed; and that on every such examination the candidates shall be examined in as many branches of general knowledge as the said Chancellor, Vice-Chancellor, and Fellows shall consider the most fitting subjects of such examination. And whereas it is expedient to extend the benefits of colleges and establishments already instituted, or which may be hereafter instituted, for the promotion of literature, science, and art, whether incorporated or not incorporated, by connecting them for such purposes with the University created by this our Royal charter—We do hereby further will and ordain, that all persons shall be admitted as candidates for the respective degrees of Bachelor of Arts, Master of Arts, Bachelor of Laws, or Doctor of Laws, to be conferred by the said University of London, on presenting to the said Chancellor, Vice-Chancellor, and Fellows a certificate from any of the institutions hereinafter mentioned, to the effect that such candidate has completed the course of instruction which the said Chancellor, Vice-Chancellor, and Fellows by regulation in that behalf shall determine.

That such certificates as aforesaid may be granted from our college called University College, or from our College called King's College, both situate in London, or from such other institution, corporate or unincorporated, as now is, or hereafter shall be, established for the purposes of education, whether in the metropolis or elsewhere within our United Kingdom, and as we, under our sign manual, shall hereafter authorize to issue such certificates.

And for the purpose of granting the degrees of Bachelor of Medicine, and Doctor of Medicine, and for the improvement of medical education in all its branches, as well in medicine as in surgery, midwifery, and pharmacy. We do further hereby will and ordain that the said Chancellor, Vice-Chancellor, and Fellows, shall from time to time report to one of our principal Secretaries of State, what appear to them to be the medical institutions and schools, whether corporate or unincorporated, in this our metropolis, or in other parts of our United Kingdom, from which either singly or jointly with other medical institutions and schools in the country or in foreign parts, it may be fit and expedient in the judgment of the said Chancellor, Vice-Chancellor, and Fellows, to admit candidates for medical degrees, and on approval of such report by our said Secretary of State, shall admit all persons as candidates for the respective degrees of Bachelor of Medicine, and Doctor of Medicine, to be conferred by the said University, on presenting to the said Chancellor, Vice-Chancellor, and Fellows, a certificate from any such institution or school, to the effect that such candidate has completed the course of instruction which the said Chancellor, Vice-Chancellor, and Fellows, from time to time, with the approval of one of our principal Secretaries of State, to vary, alter, and amend any such reports, by striking out any of the said institutions or schools included therein, or by adding others thereunto.

That the said Chancellor, Vice-Chancellor, and Fellows, shall have power, after examination, to confer the several degrees of Bachelor of Arts, Master of Arts, Bachelor of Laws, Doctor of Laws, Bachelor of Medicine, Doctor of Medicine, and to examine for medical degrees in the four branches of medicine, surgery, midwifery, and pharmacy, and that such reasonable fees shall be charged for the degrees so conferred as the said Chancellor, Vice-Chancellor, and Fellows, with the approbation of the Commissioners of our Treasury, shall from time to time direct; and such fees shall be carried to one general fee fund for the payment of the expenses of the said University, under the directions and regulations of the Commissioners of our Treasury, to whom the accounts of income, and expenditure of the said University, shall once in every year be submitted, which accounts shall be subject to such examination and audit as the said commissioners may direct.

That at the conclusion of every examination of the candidates, the examiners shall declare the name of every candidate whom they shall have deemed to be entitled to any of the said degrees, and the departments of knowledge in which his proficiency shall have been evinced, and also his proficiency in relation to that of other candidates, and he shall receive from the said Chancellor a certificate, under the seal of the said University of London, and signed by the said Chancellor, in which the particulars so declared shall be stated.

Provided always, that all by-laws and regulations made from time to time touching the examinations of candidates, and granting of degrees, shall be submitted for the consideration of one of our principal Secretaries of State, to be approved of by him.

And lastly, we do hereby, for us, our heirs and successors, grant and declare that these our letters patent, or the enrolment or exemplification thereof, shall be in and by all things valid and effectual in law, according to the true intent and meaning of the same, and shall be construed and adjudged in the most favorable and beneficial sense for the best advantage of the said University, as well in all courts as elsewhere, notwithstanding any nonrecital, misrecital, uncertainty, or imperfection, in these our letters patent.

In witness whereof, we have caused these our letters to be made patent. Witness ourself, at our Palace of Westminster, the 28th day of November, in the seventh year of our reign.

By Writ of Privy Seal, EDMUNDS.

---

